# Creating Expressive Social Robots That Convey Symbolic and Spontaneous Communication

**DOI:** 10.3390/s24113671

**Published:** 2024-06-05

**Authors:** Enrique Fernández-Rodicio, Álvaro Castro-González, Juan José Gamboa-Montero, Sara Carrasco-Martínez, Miguel A. Salichs

**Affiliations:** RoboticsLab, Department of Systems Engineering and Automation, Universidad Carlos III de Madrid, Av. de la Universidad 30, 28911 Madrid, Spain; acgonzal@ing.uc3m.es (Á.C.-G.); jgamboa@ing.uc3m.es (J.J.G.-M.); sacarras@ing.uc3m.es (S.C.-M.); salichs@ing.uc3m.es (M.A.S.)

**Keywords:** expressiveness management, multimodal interaction, human–robot interaction, affective state expression

## Abstract

Robots are becoming an increasingly important part of our society and have started to be used in tasks that require communicating with humans. Communication can be decoupled in two dimensions: symbolic (information aimed to achieve a particular goal) and spontaneous (displaying the speaker’s emotional and motivational state) communication. Thus, to enhance human–robot interactions, the expressions that are used have to convey both dimensions. This paper presents a method for modelling a robot’s expressiveness as a combination of these two dimensions, where each of them can be generated independently. This is the first contribution of our work. The second contribution is the development of an expressiveness architecture that uses predefined multimodal expressions to convey the symbolic dimension and integrates a series of modulation strategies for conveying the robot’s mood and emotions. In order to validate the performance of the proposed architecture, the last contribution is a series of experiments that aim to study the effect that the addition of the spontaneous dimension of communication and its fusion with the symbolic dimension has on how people perceive a social robot. Our results show that the modulation strategies improve the users’ perception and can convey a recognizable affective state.

## 1. Introduction

Robotics is a field that has been experiencing a continuous growth in recent years. Robots have been completely integrated into sectors such as industry, and we are starting to see robots in areas that involve interacting directly with people. Examples include healthcare [1], where patients require 24/7 assistance, or guiding people through public places, such as museums [2] or shopping malls [3]. In these scenarios, it is desired that robots interact with humans in a way that feels natural. According to research [4], interactions between humans and computers are fundamentally social, as humans tend to use social responses regardless of how human-like the computers might appear. Based on this, drawing inspiration from social psychology or communication theory might be beneficial when designing robots for tasks that involve human–robot interaction. The design of the robot’s appearance and social behaviour can enhance factors like interpersonal trust and companionship appearance or decrease psychological reactance [5,6,7,8].

When endowing a robot with communicative skills, there are several aspects of communication that should be considered. In 2002, Buck and VanLear [9] proposed that human communication can be decoupled into two components: symbolic and spontaneous communication. While the former deals with conveying communicative messages to the other peer in the interaction, the latter modulates the message in order to reflect the speaker’s motivational and/or emotional state. For example, if a person tells a friend that she/he bought a new car, the words uttered would represent the symbolic component, as they seek to achieve a communicative goal (informing about the car purchase). Meanwhile, the spontaneous component would be carried by factors like the pitch of her/his voice or her/his body language, as they might transmit her/his happiness or excitement over the purchase. Based on this knowledge, it can be assumed that it would be beneficial for social robots to combine both components of communication in their expressiveness. This might help to ease the creation of bonds between users and the robot, which improves the overall quality of the interaction.

Endowing a social robot with the ability to use symbolic communication entails generating actions for each of the robot’s actuators and combining them into multimodal messages. The information conveyed has to be coherent across all modalities (i.e., all interfaces seek to achieve the same communication goal). The morphology of the robot (i.e., the number, type, and placement of the robot’s actuators) is another factor that needs to be taken into account, as it will constrain the ways in which the robot will be able to express itself. Also, the messages have to abide by the constraints of real interactions. An example of this would be the maximum delay that can exist between asking a question and receiving the answer. Finally, the robot’s expressiveness should be adapted to different factors related to the interaction, for example using a greeting adapted to the identity of the person interacting with the robot, even though a generic greeting could achieve the same goal. In this work, we refer to the set of factors that are external to the robot and play a role in an interaction as the context of the interaction. This includes factors such as the time of day, the location of the robot, or the identity of the user interacting with the robot (among others).

Regarding the spontaneous dimension of communication, one of the internal states that has attracted the largest amount of attention in human–robot interaction is the emotional state. This component of communication plays a key role in interactions between persons. Emotions are functional processes that can coordinate and adapt social interactions [10]. They also help to regulate and bias processes, contributing to intelligent functioning [11]. Emotional expression has been shown to provide solutions to relational problems, such as initiating and maintaining reciprocity, hierarchy, trust, and fitness. When applied to robotics, research has shown that people can be more inclined to interact with an artificial agent that is able to clearly express emotions [12]. Users seem to show a preference for robots that are able to use emotional expressions (in this work, we will use *gesture* and *expression* indistinctively for a combination of multimodal actions aimed at conveying a particular communicative goal) and can display positive affective responses to them [13].

If we apply the theory of communication proposed by Buck and VanLear to the field of robotics, the task of endowing a robot with a natural expressiveness entails three distinct problems:

- **Problem 1:** How to use expressions to convey information oriented to achieve a specific communicative goal. This would correspond to the symbolic dimension of communication. It is a complex task that requires designing a model for representing these expressions, managing the different actuators of the robot to perform the actions indicated by the model, and synchronising these actions to create cohesive multimodal messages that seem natural to the user.
- **Problem 2:** How to express in a perceivable way the motivational/emotional state of the robot when interacting with users. Because the robot’s state has to be perceived continuously, it is not enough to use punctual expressions to convey it. Instead, this problem entails finding a solution for adapting the entire expressiveness of the robot to its internal state.
- **Problem 3:** How to fuse both components of communication in a way that makes the robot’s state perceivable while conveying task-related (symbolic) information, without hindering the interaction. This means that the user interacting with the robot needs to be able to properly recognise the communicative goal behind the symbolic dimension of the information received, while still being able to properly identify the robot’s internal state.

With this manuscript, our goal is to present our solution to the three problems presented above, as a way to validate the importance of a proper fusion of the symbolic and spontaneous dimensions of communication. We seek to find the effect that the different components of communication have on the users’ subjective perception of the robot. Also, we consider that this perception is the main indicator of how our solution performs. Because of this, these problems cannot be tackled from an objective, mathematical perspective. Instead, we focused on subjective evaluations based on questionnaires to determine how users see our robots in terms of warmth, competence, and discomfort. In this paper, the first contribution is the design of a method rooted in Buck and VanLear’s communication theory to convey the spontaneous and symbolic dimensions of communication, while decoupling both dimensions. This allows developers to focus on designing the task-related expressions (symbolic dimension), while the system takes care of conveying the robot’s state (spontaneous) through the use of three modulation strategies. The proposed approach allows multimodality for both dimensions, and fuses different internal states (in particular, mood and emotion). In order to achieve this, our second contribution is the development of an expressiveness system that relies on a library of predefined expressions to create the symbolic dimension of communication. This module, along with our dialogue manager [14], forms the human–robot interaction that has been integrated and tested in our robotic platforms. In order to adapt the robot’s symbolic communication to the context of the interaction, and mitigate the potential issue of repetitive expressiveness due to relying on predefined expressions, we have integrated in this expressiveness architecture three techniques for dynamically adapting our robot’s expressiveness. The first one allows developers to specify changes that have to be made to the expression before executing it. For example, the robot could be asked to use a generic greeting gesture, but replacing the utterance by a new one that takes into account the identity of the user. While this adds variability to the robot’s expressiveness, it still requires developers to manually code the actions of the robot. The other two techniques are oriented to overcome this limitation: (i) a paraphrasing module that rephrases the utterances of the robot without changing the meaning or adding new information, presented originally in [15]; (ii) a module for adapting the verbal messages uttered by the robot to the user (for example, rephrasing an explanation into a simplified version if the robot is talking to a child), as seen in [15]; and (iii) a module for automatically selecting appropriate non-verbal gestures from a library that suit the robot’s speech, introduced first in [16].

When considering the spontaneous dimension of communication, in this work, we have focused on conveying the robot’s mood and emotions, due to the important role that they play in interaction. While emotions are short-lived and more intense, moods extend longer in time, and their effect on expressiveness is more subtle [17]. For systems using predefined gestures, affective expression can be achieved by developing different gestures for each possible state. However, this leads to scalability issues, as developers need to create multiple expressions to achieve the same goal. A solution for this is to model expressions without taking the spontaneous dimension into consideration and, then, modulate them so they display the state that has to be conveyed [18,19]. In this work, we focus exclusively on the expressiveness aspect of affect display (thus, the generation of the affective states is outside of this work’s scope). We have decided to follow the second approach and implemented two modulation strategies that can be used for altering the gestures to display the affective state of the robot. The first strategy, which was presented originally in [20], modifies the overall appearance of a gesture through the use of two global parameters: speed and amplitude. These parameters will have a different effect on each of the robot’s interfaces (e.g., for the motions, the parameter speed modifies the speed of the motors, while for the eyes, it modifies the blinking speed). The second strategy uses modulation profiles that specify the changes that each of the robot’s interfaces should experience depending on the robot’s affective state. For example, the profile might indicate that the robot being happy translates into a higher pitch of the voice and faster motions. The last contribution of this work is the evaluation of the effect that the combined display of the spontaneous and symbolic dimensions of communication has on how people perceive a social robot, when compared with a robot that only displays one or the other. This last contribution corresponds to the evaluation stage of the solution to the problems presented above.

The rest of this manuscript is structured as follows. Section 2 presents a review and comparison of the state-of-the-art in the field of expressiveness generation. In Section 3, we introduce Mini, the robotic platform in which our approach has been integrated, and present the Expression Manager, which is the module in charge of controlling the robot’s expressiveness features. The modulation strategies that have been developed for adapting Mini’s expressions to the context of the interaction and for conveying the spontaneous aspects of interactions are presented in Section 4. In Section 5, we provide the results of an evaluation performed to prove the capabilities of our solution, along with the limitations of this approach. Finally, we present the conclusions extracted from our work in Section 6.

## 2. Related Work

In this work, we will refer to *expressiveness generation* as the method for endowing robots with the ability to convey the symbolic and/or spontaneous components of communication. We have decided to classify expressiveness-generation approaches into two main categories: (i) works that rely on libraries of predefined expressions and (ii) works that generate the robot’s response from scratch. First, we analysed a series of relevant works from each of these two approaches. Then, we compared these studies to each other and to our approach, in order to highlight the innovative aspects of our system. This comparison has been made based on three features that we consider to be important for an expressiveness system. The characteristics that we selected are as follows:

- **Gesture design:** How the gestures are modelled, as well as the synchronisation method for all the modalities in the expressions. This feature is connected to the first of the three problems described in the previous section.
- **Multimodality:** Modes of interaction that the expressions are able to use. This feature is connected to the first of the three problems described in the previous section.
- **Adaptability:** If the proposed approach is able to adapt its gestures to different conditions (e.g., affect states). This is connected to the second and third problems described in the previous section.

### 2.1. Gesture Design

When analysing the works in the literature depending on how their systems model the robot’s expressiveness, we can see a marked difference between authors that use predefined expressions and those that generate the gestures dynamically. Among the latter, there is a small variation on how their systems create expressions, with most of these using the audio of the speech for creating the expressions. For example, Hasegawa et al. [21] proposed using a bi-directional LSTM neural network that receives feature vectors extracted from the robot’s speech wave, and generates 3D positions. Kucherenko et al. [22] presented an encoder–decoder system that learns to transform motions into low-dimensional representations and back to motions. They then replaced the encoder with a new version trained to convert speech into the same low-dimensional representation. This way, the robot’s speech is encoded into a low-dimensional space, and then decoded into motions. The system proposed by Ginosar et al. [23] starts by learning a mapping from speech to motions. Then, it uses an adversarial discrimination method for ensuring that the generated gesture matches the typical motion of the speaker, and that there is no regression to the mean of all possible gestures.

Other authors have opted for extracting information from the transcription of the speech for gesture generation. Among these, Yoon et al. [24] proposed a sequence-to-sequence approach where the encoder processes the speech and the decoder generates motions. Both the encoder and decoder are modelled with recurrent neural networks. A soft attention mechanism ensures that the decoder focuses on specific keywords, instead of on the whole text. For long speeches, the network is inferred multiple times and the sequences of gestures are then concatenated. The work of Aly and Tapus [25] uses handcrafted rules to generate a set of gestures based on the robot’s response to the user. Metaphoric gestures are generated separately. While this approach uses the transcription of the speech for gesture generation, the speech itself is generated taking into account the personality of the user (introverted/extroverted). The system proposed by Ahuja and Morency [26] in 2019 separates itself from the rest by not generating expressions for accompanying the speech, and instead generating gestures based on textual descriptions. Their approach uses a curriculum learning neural architecture to learn a joint embedding of language and pose, and generating new sequences of poses. This method emphasises shorter and easier sequences. The proposed approach uses an LSTM network to encode the speech, and a GRU network to decode poses. There are also works that combine both audio and text-based features of the speech for gesture generation. For example, Ravenet et al. [27] presented a model to generate gestures for virtual agents based on the linguistic structure, the prosodic information, and the meaning conveyed by the agent’s speech. The process begins by identifying the Image Schemas (i.e., recurring patterns used to map conceptual representations of the world from one domain to another) that are present in the agent’s speech. Gesture invariants tied to the identified Image Schemas are used to build the corresponding gestures, which are then combined into the final behaviour.

Finally, one last group of authors have used other types of information alongside the robot’s verbal message for generating expressions. Among these new sources of information, one that has been widely used is the affective state of either the agent or the user. For example, Spitale and Matarić [28] presented an approach that analyses the transcription of the robot’s speech and extracts three features: (i) the timing and duration of the words; (ii) the valance, arousal, and dominance for each word; and (iii) an Image Schema representing the iconic meaning of the text. The system takes only words with associated Image Schema mappings and emotional states, along with their speech-timing information. A gesture shape extractor selects the shape of the expression associated with each word from its associated Image Schema, while a gesture parameter extractor uses the affective state to define the gesture’s speed, timing, and amplitude. Based on these gestures’ affective state and meaning in the context of the sentence, a gesture ranker selects one gesture to be generated. Zabala et al. [29] presented a method for adapting beat gestures, lights in the eyes, body posture, and vocal intonation and volume to the sentiment of a humanoid robot’s speech. The system first retrieves the valence score for each word in the script using the *VADER* sentiment analyser. Then, it generates body gestures using a Generative Adversarial Network based on the duration of the speech. Finally, the gestures generated are adapted based on the valence values. The authors also propose that the robot’s personality should change how constrained or exaggerated the generated behaviours are. The solution proposed by Qi et al. [30] takes into account the emotion of the agent. The first module in the architecture, the Emotion–Beat Mining module, extracts emotional and audio beat features from the robot’s speech audio. A Spatial–Temporal Prompter module then takes the initial pose of the agent, and generates future poses, ensuring smoothness via prompt enhancement. For this, they use spatial interpolation and temporal-reinforcement prompt learning. Rawal et al. [31] presented ExGenNet, a deep generative approach for creating facial expressions for a humanoid robot. This system combines a generator network trained for converting joint angles into a simplified image of facial features, and a classifier that maps those images into discrete predefined expressions (e.g., “angry”, “happy”, “neutral”, “sad”, and “surprised”). By performing grid search and gradient descent, the system finds the joint configurations that generate a particular facial expression. The work of Auflem et al. [32] also sought to control facial expressions in a humanoid robot. Their system identifies the robot’s facial action units (AUs) through a computer vision analysis, and learns the mapping between these AUs and the actuation commands by introducing random actions and observing the changes in the AUs. Another input that has been widely considered in the field of gesture generation is the target speaker’s gesticulation style. In 2022, Ahuja et al. [33] proposed a method for adapting a generalised gesture-generation method to a specific gesticulation style for which there is a low amount of training data. For this, the model is taught first to identify the cross-modal grounding shifts by changing the loss function. Then, a second loss function is added to encourage the model to shift its output domain so it is closer to the target’s. Fares et al. [34] presented a model for modelling behaviour style for embodied communicative agents based on the speech’s audio and transcript. An encoder network analyses both input features and upper-body gestures and uses that information for modelling a speaker’s behaviour style. Then, a sequence-to-sequence synthesis network conditioned by the target speaker’s style generates motions based on the two inputs considered. Finally, a fader network is in charge of disentangling style and content from the multimodal data.

A bigger variation on gesture design approaches can be found among the methods that rely on a predefined set of expressions. This is the approach that we have followed in this work. Meena et al. [35] proposed a system for a Nao robot that uses the number of words in the utterance to align the stroke phase of the gesture with the speech. The stroke phase refers to the part of the gesture where the meaning is conveyed. The system then interpolates introductory and retraction poses with the stroke phase to generate an expression with an appropriate length. Xu et al. [18] developed an expressive model where task-specific features of expressions are defined in handcrafted profiles. Non-task-related features, in particular mood expression, are conveyed through a set of modulation parameters. Glas et al. [36] presented an overview of the conversational android ERICA. Its dialogue manager selects the appropriate sequence of utterances, gestures, and facial expressions as a response to a user utterance. The non-verbal messages can be used to convey mood and back-channelling or to simulate basic human behaviours (e.g., breathing or blinking). Ribeiro et al. [37] presented the *SERA* ecosystem, a combination of a series of tools and a model for using AI agents with robotic embodiments in human–robot interaction scenarios. A behaviour planner receives multimodal instructions combining non-blocking markups (i.e., can be executed in parallel) for the TTS and non-verbal gestures. An animation engine decides which behaviours are performed and how the different modalities are blended, and executes them. Behaviours can be designed through this engine or with animation tools. Groechel et al. [38] proposed using augmented reality to enhance the capabilities of robots with low expressiveness, by adding mixed-reality arms in an armless tabletop robot, Kuri.

Gomez et al. [39] presented the expressiveness system for the Haru tabletop robot, which uses movable LCD screens for eyes as its main means of communication. They followed the 12 principles of animation to create multimodal gestures. Each modality was first designed separately and then combined either manually or through an automatic process that ensures physical feasibility, while maintaining modality synchronisation. Hong et al. [40] proposed a multimodal interaction architecture that adapts the robot’s state to the user’s affective state. This is achieved through the combination of an affect-recognition module, an emotional state-generation module, and a behaviour-selection module. Each possible emotion has multiple expressions associated with different intensity levels, and the robot combines them with task-related utterances. The approach of Suguitan et al. [19] uses a variational autoencoder and an emotion classifier for modulating neutral expressions into affective expressions. Asai et al. [41] also proposed adapting the robot’s responses to its emotional state. The system first uses BERT to select the predefined answer that better works as a response to the user’s utterance. Then, it selects the appropriate response variant based on the robot’s emotional state. Handcrafted non-verbal gestures are selected based on the robot’s emotion, and repeated based on the duration of the response. Sönmez et al. [42] presented an artificial empathetic two-layer system, where the first layer maintains the robot’s internal state, and the second controls the robot’s flow of emotions. This flow is affected by the robot’s current emotion, its internal state and mood (updated based on the last five emotional states), and the user’s perceived emotion.

### 2.2. Multimodality

If we evaluate the expressiveness channels that the different approaches use, we see that almost all of them use at least speech and body motions. If we divide the works again based on the strategy followed to generate the expressions (i.e., handcrafted or generated at runtime), then we see that most of the works that generate gestures at runtime are systems that use different features of the robot’s speech to generate body motions. This is the case for the works of Hasegawa et al. [21], Ravenet et al. [27], Kucherenko et al. [22], Ginosar et al. [23], Yoon et al. [24], Yoon et al. [43], Spitale and Matarić [28], Ahuja et al. [33], Fares et al. [34], and Qi et al. [30]. Among the works that use handcrafted expressions, there are also several authors that focused exclusively on the combination of speech and body motions and posture, like Meena et al. [35], Xu et al. [18], or Asai et al. [41]. There are also authors that only considered body motions and postures, without the verbal component. Examples can bee seen in the works of Suguitan et al. [19] or Groechel et al. [38]. The latter approach is unique in that it uses augmented reality as part of the robot’s expressiveness, complementing the physical actions of the platform.

Besides speech and motions, the next-most used expressiveness modalities have to do with facial expression, either through changes in the robot’s gaze or by changing the actual expression in the robot’s face. Examples of the first can be seen in the approaches presented by Ribeiro et al. [37], Aly and Tapus [25], or Gomez et al. [39]. These three authors combined gaze expression with other modalities like speech or gestures. The work of Gomez et al. is particularly interesting, as the eyes are the main expressive feature in their robot, moving them and displaying different gazes. Other authors, like Rawal et al. [31] or Auflem et al. [32], focused on altering the robot’s facial features to convey different expressions. Finally, Glas et al. [36] combine both gaze and facial expression control in their approach. Another interface that is present in multiple robots is coloured LEDs, usually combined with other modalities. This can be seen in the approaches of Gomez et al. [39], Hong et al. [40], or Zabala et al. [29].

While speech, motions, facial expressions (including gaze), and LEDs are the main modalities used in the works reviewed, there are also other channels that have been explored. For example, Sönmez et al. [42] included a touch screen that the robot would use to display emojis that represented its internal state. Finally, Klausen et al. [44] proposed using different breathing patterns in a soft robot to convey emotions, by inflating and deflating the body of the robot.

### 2.3. Adaptability

Finally, we see that the approaches that generate the expressiveness automatically present a high adaptability, given that most of the methods reviewed not only use the text of the speech, but also the prosodic features. This is interesting for adapting the robot’s expressions to different internal states because, if these states alter either the content of the speech or the prosody of the voice (among other communication modalities), then this will also result in a change in the gestures being created. Examples of approaches that rely either on prosodic and/or semantic features can be seen in the works of Hasegawa et al. [21], Kucherenko et al. [22], Ginosar et al. [23], or Yoon et al. [24]. The approaches that use a library of predefined gestures show less adaptability, because the whole expressiveness of the robot has to be designed beforehand. However, authors have proposed different solutions for overcoming this issue. For example, the work of Meena et al. [35] allows adapting the length and amplitude of the gestures used based on the utterances they will accompany.

Among the authors that have proposed strategies for adapting an agent’s expressiveness to different factors, a large portion of them focus on how to convey the agent’s affective state (i.e., moods and emotions). Some authors opted for using different versions of handcrafted expressions for conveying each possible emotion. For example, Hong et al. [40] presented a system that included both deliberative and reactive emotions, both modelled as discrete values. The former are updated based on the recognised state of the user and on interaction responses, while the latter are determined based on environmental stimuli. The proposed system then selects between both sources of emotions based on a priority system and executes the emotional expressions associated with each source. The approach of Asai et al. [41] updated the robot’s emotional state using a logistic regression model that takes the previous state and the dialogue history. Their system not only considers different emotional expressions, but also emotional versions of the robot’s utterances. In the work of Sönmez et al. [42], the robot’s flow of emotions is affected by its mood and the user’s emotion.

Other authors opted for designing solutions that allow modulating the robot’s expressions, so they can convey different affective states with the same gesture instead of designing multiple versions of the expressions. Xu et al. [18] proposed separating those features of expressions that are task-specific and those that are task-independent, and used the latter to convey emotions. For this, the gesture function (e.g., waving) is selected based on the task at hand, and then parametrised based on the robot’s mood. Among the parameters that can be configured, we can find the amplitude, hand height, finger rigidness, repetition, hold time, or decay speed, among others. Suguitan et al. [19] proposed an architecture for converting neutral expressions into emotional gestures. An autoencoder compresses the original motion into a lower dimensional space, while the emotion classifier extracts its arousal and valence. The new emotional state is conveyed through a modulation of these two parameters, and the low-dimensional representation is decoded into a new gesture. Spitale and Matarić [28] proposed a system that extracts the valence and arousal of the words in the robot’s speech, and uses these values to modulate the timing and amplitude of the gestures generated. In their system, the shape of the gesture is connected to the semantic information in the speech. A gesture ranker then selects one expression per sentence, among all of the ones generated. Zabala et al. [29] also extracted the sentiment of the robot’s speech. This value is simultaneously passed as an input to the GAN-based gesture-generation method, converted into RGB values for the LED in the robot’s eyes, and used to control the volume, speed, and pitch of the robot’s voice. Finally, both Rawal et al. [31] and Auflem et al. [32] proposed solutions for conveying emotion via facial expressions. Rawal et al. proposed generating joint configurations for the facial actuation units, classifying the resulting face into one of five emotions, comparing this emotion with the desired one, and using the error to optimise the generation process. In the work of Auflem et al., the robot’s face is analysed using a Residual Masking Network (RMN) to evaluate the facial expressions of emotion, with the goal to improve the robot’s ability to convey emotions.

Finally, although affective state is one of the most prevalent factors considered when modulating a robot’s expressiveness, there are other factors that also play a role. For example, the solution proposed by Aly and Tapus [25] takes into account the user’s personality (introverted/extroverted) when generating the response that the robot will give to a utterance, and then generates the expressions according to this response. For the metaphoric gestures, the amount of expressions performed and the amplitude of the motion curves is adapted to the level of introversion/extroversion. For the rest of the expressions, a series of modulation parameters is used to adapt them. Another example is the work that Yoon et al. [43] presented in 2020, where the identity of the user is passed alongside the robot’s speech to the gesture-generation system to produce appropriate expressions. Another factor that is considered by many authors is the style of gesticulation of a speaker. For example, Ahuja et al. [33] tried to solve the issue of having a low amount of training data for the desired gesticulation style by adapting the behaviour of a generalised gesture-generation method. Fares et al. [34] proposed modelling the speaker’s style from his/her upper-body gestures and speech audio and transcript, and to use that to condition the generation of new gestures.

### 2.4. Comparison with Our Approach

The approach that is presented in this manuscript finds its roots in the theory of communication proposed by [9]. This theory considers that human communication can be divided into two dimensions: the symbolic dimension carries the message that has to be conveyed, while the spontaneous dimension transmits the emotional and motivational state of the speaker. The main difference between our work and almost all the solutions reviewed is the proposal of a multimodal system that decouples these two dimensions. This enables designers to focus on the symbolic messages, while the system controls the spontaneous dimension. While most of the approaches discussed in this section also implement both dimensions, decoupling them gives developers a high degree of control over how the robot communicates task-related information, without having also to design the spontaneous component (although having the option of doing so). Of the works reviewed in this section, the solution presented in this manuscript is closer to the one presented by [18] (one of the few that also considers this decoupling of components), as we also modified predefined expressions using non-task-related features (i.e., features not involved in conveying the expression’s communicative goal). However, our solution complements this with two other approaches: (i) replacing individual actions in the expression (e.g., changing the sentence uttered by the robot) and (ii) creating modulation profiles to define the effect that each of the robot’s states should have on the expressions (e.g., happiness produces a raising of the pitch of the voice and the speed of the movements). Regarding the internal states considered, our work focuses on affective states, similar to the approaches presented by [19,28,41,42]. However, while Suguitan et al. used machine learning to adapt the expressions, we considered that the amount of possible states (four emotions, four moods, and a neutral state) is low enough that handcrafting the parametrisation would not be an excessive burden for the roboticists, and would give them more control of the result of the modulation. Regarding the work of Asai et al. and Sönmez et al., the difference is that those approaches use predefined expressions to convey affective states, while we rely on modulating our expressions. This enhances the scalability of the system, as we would not have to create different versions for each affective state considered every time we want to add a new gesture to the library. Finally, compared with the work of [28], our approach allows for a more in-depth modulation because we can alter individual parameters of the different interfaces (i.e., the pitch of the voice or the intensity of the robot’s LED), instead of altering the expressions’ general speed, amplitude, and timing.

Regarding gesture design, our approach is similar to the work presented by [37], as we also define the different modes of communication and their combination explicitly. However, our approach discards the use of markup languages and, instead, uses state machine-like structures. This simplifies the process of creating and connecting all communication modalities simultaneously and introducing structures like loops or the conditional execution of states to simplify the creation of gestures. Finally, if we focus on multimodality, our work is comparable to the one proposed by [39], as we use similar interfaces. The two differences are as follows: (i) in our approach, all modalities can be designed simultaneously and be interwoven from the start, while the approach proposed by Gomez et al. defines each modality independently, and then synchronises them; (ii) our system uses a touch screen to display images or icons as part of the multimodal messages.

## 3. Designing an Expressiveness Architecture Based on Predefined Gestures

**Figure 1:**
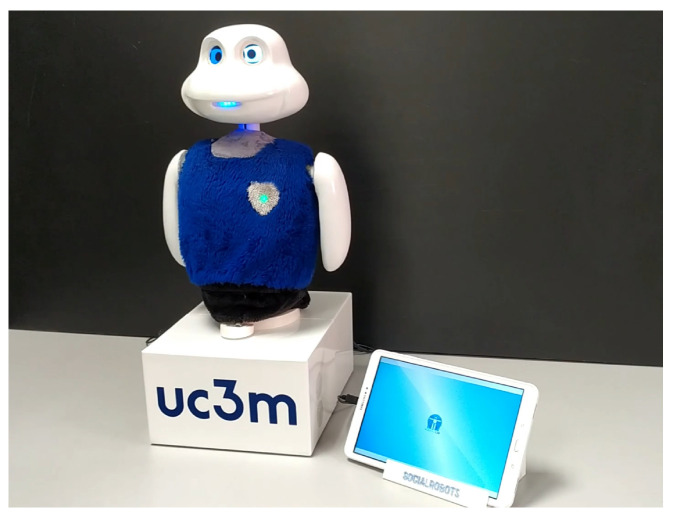
Mini, a social robot developed for interacting with adults who are older that suffer from mild cases of cognitive impairment.

The expressiveness model that we presented in the previous section has been integrated into the Mini social robot [45]. Mini, shown in Figure 1, is a tabletop robot with a friendly appearance that was designed with the idea of serving as a 24/7 companion for adults who are older who have mild cognitive impairment. It has five degrees of freedom (two in the neck, one for each shoulder, and one in the base), a coloured LED that lights the heart of the robot, two OLED screens as eyes, and a touch screen to display multimedia content and also to communicate with the user through menus.

**Figure 2:**
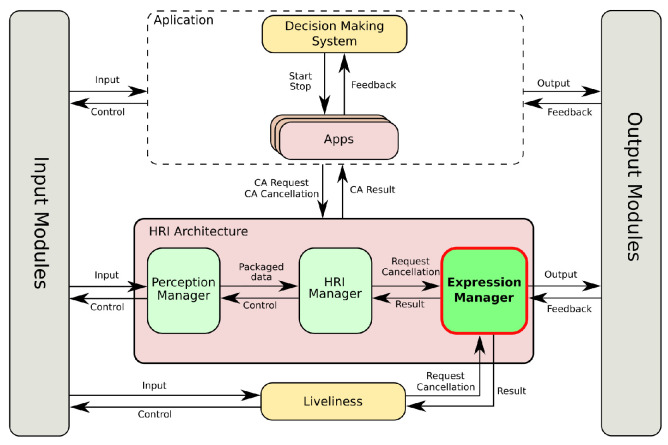
Software architecture integrated in Mini.

Mini can play games (e.g., quiz games or bingo), show multimedia content to the user (e.g., pictures, films, music, etc.), and also propose cognitive stimulation exercises. While the robot was created for assisting adults who are older, its software architecture [45] was designed for general interactions, so it could also be used in other contexts. This architecture, as depicted in Figure 2, was designed following a modular approach and can be divided into two levels, as well as three modules that are transversal: the input and output modules are in charge of controlling the robot’s sensors and actuators, respectively, while the context serves as the robot’s memory, both short- and long-term. At the top level, we find the Decision-Making System (DMS) and the applications. The applications provide all of the tasks that Mini can perform (e.g., play a game, read the news to the user), while the DMS controls which application has to be activated at any time.

The lower level in Mini’s software includes the following elements. The Liveliness module generates non-task-related expressions to give the robot a lively appearance. The Perception Manager packages and formats the information captured by the sensors. The HRI Manager controls the flow of interactions using information coming from the Perception Manager. Finally, the last module in the architecture, and the main focus of this manuscript, is the Expression Manager.

### The Expression Manager

**Figure 3:**
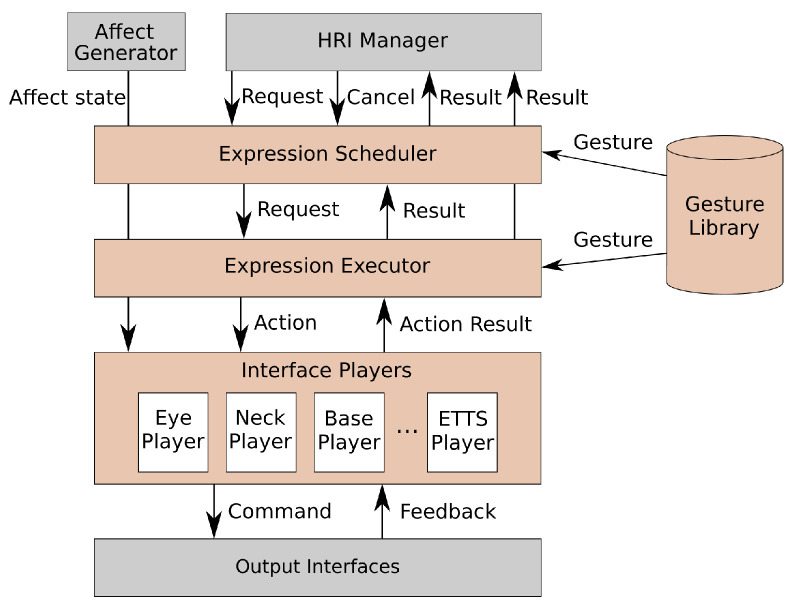
Overview of the Expression Manager. The blocks in grey (i.e., affect generator, HRI Manager, and output interfaces) are outside of this work’s scope.

The Expression Manager, shown in Figure 3, is our solution to the three problems described in Section 1. It is in charge of orchestrating all the expressive capabilities of the robot. It receives requests for gesture execution, and performs the following four tasks:

1. Keeps track of the interfaces that will be required by any requested expression, as well as the interfaces of the robot that are already being used by one of the active gestures. This ensures that no interface is being used by more than one expression at a time.
2. Manages the priority of the expressions requested, storing them in priority queues and executing them from higher to lower priority.
3. Displays the internal state of the robot at any given time by properly modulating the robot’s expressiveness.
4. Connects the rest of the software architecture with the modules that control the robot’s actuators by transforming the actions defined in the expressions into commands that these modules can understand.

The Expression Manager in turn is divided into the Expression Scheduler (in charge of the first two tasks mentioned above), the Expression Executor (collaborates with the Scheduler in conducting the second task), and the Interface Players (in charge of the third and fourth task mentioned above). The Expression Scheduler receives gesture execution requests that contain the name of the expression and its priority level, plans the execution of these expressions, and ensures that no conflicts arise between them. These conflicts involve the new gesture requiring the use of an interface that is currently being used by an expression that is already in execution. In the event of a conflict arising, the Expression Scheduler compares the priority of the expressions involved and decides which ones should be performed immediately, which ones should be stored to be performed once the required interfaces are free, and which ones should be directly discarded. The Expression Scheduler can also receive requests to stop active gestures.

For defining the priority that an expression should have, the majority of gestures required by the apps and the current interaction will have medium priority by default. Gestures that require immediate execution regardless of what the robot is doing will have high priority. Examples of this would be expressions for reacting immediately to a sudden event, like complaining if the user hits the robot. Finally, low priority will be assigned to gestures that are used for enhancing interactions, but that do not seek to achieve any particular communicative goal. When evaluating conflicts between expressions, the one with the highest priority will be executed (if the gesture with the highest priority is the new one, the active one will be stopped immediately). The other will be sent to the priority queue to be executed once the interfaces are free (if it has medium priority) or discarded completely (if it has low priority). When an expression is ready to be executed (either because the interfaces are free or because it has a higher priority), the Expression Executor is the module of controlling this execution process. When the Scheduler sends an activation request for an expression, the Executor searches through the gesture library, configures it based on the information provided in the activation request, and starts its execution.

In our architecture, gestures are represented as state machines, using a modified version of *FlexBE* [46], a framework for developing robot behaviours using a graphic interface (GUI). The original version of this framework was designed for managing high-level robot behaviours, and was limited to having a single machine running at any given time. We modified it to allow for parallel execution of expressions, eliminate features that our architecture would not need in order to speed up the execution process, modified the GUI so all the parameters common to all expressions are initialised by default, and added a new gesture template from which all behaviours will inherit that provides features required by all expressions. Our framework is also able to create an expression dynamically by receiving a list of actions from the applications and generating the corresponding FlexBE state machine at runtime. The use of *FlexBE* allows people without a technical background to create expressions thanks to the GUI, while the addition of a method for generating an expression dynamically from a list of actions frees developers from having to go through the process of manually creating each state machine.

The final layer in the Expression Manager includes the Interface Players. These are the modules that interact directly with the robot’s output interfaces. There will be an individual player for each of the robot’s actuators. In the case of Mini, we have the following players: one for each of its five joints, one for the eyes, another two for the LEDs in the heart and the cheeks, a player for the voice, and one last player for the touch screen. All of the players have been developed using the same template, based on *ActionLib* (https://wiki.ros.org/actionlib (last accessed on 25 May 2024)). Among the features the players provide are the following: (i) they can be individually enabled/disabled; (ii) they provide feedback about the execution of the different actions and notify about their completion; (iii) they can cancel actions at any given time.

Each expression is a combination of multimodal actions. Whenever each of these actions has to be performed, it is sent to the corresponding player (for example, if the robot has to utter a sentence, it will be sent to the ETTS Player). Each player can perform only one action at a time (it is impossible for a joint to reach two positions at the same time, for example). The Player receives the action that has to be performed, and prepares the command that the corresponding interface can understand. This includes using knowledge from the robot’s context module to complete missing information. For example, if a gesture wants to use the user’s name, then it can use a specific tag (@name in this case), and the Player will replace it with the appropriate value. The Players can also manage unexpected situations, in case the corresponding interface crashes. Whenever the action is completed, the Player notifies that the interface is available once again and returns the result of the action.

Every time the execution of an active gesture is completed, the Scheduler looks for the next expression with the highest priority that can be performed. If a gesture is found, the Executor loads it and executes it. If no gesture can be performed (because the required interfaces are not available), or there are no queued expressions, then the Scheduler waits until the next gesture request arrives. Finally, the Expression Manager can receive requests for stopping a given expression at any time. When one of these requests is received, first, the Scheduler checks if the expression is being performed (in which case, the Expression Executor stops it), or if it is scheduled to be performed once the interfaces are available (in which case, the Scheduler discards it).

While the architecture described here was first designed to convey the symbolic component of communication, it was later extended with the capabilities for conveying the spontaneous dimension. This is achieved through the use of a series of modulation techniques that allow the expressions designed using *FlexBE* to be adapted at runtime. These techniques will be presented in the next section.

## 4. Dynamic Expression of Symbolic and Spontaneous Aspects of Communication

The Expression Manager, which was presented in Section 3, endows our robots with the ability to convey the symbolic dimension of communication, that is to achieve different communicative goals. However, the solution proposed poses two main issues. On the one hand, using predefined expressions can cause the symbolic messages conveyed to feel repetitive to the users interacting with the robot. On the other hand, as argued in Section 1, it is also necessary to make the robot able to convey the spontaneous dimension of communication, in order to achieve a natural interaction. In this work, we propose the use of three modulation techniques to tackle these two issues. The first method seeks to enhance the robot’s ability to convey the symbolic dimension of communication by adapting the expressions used to the context of the interaction (Section 4.1). The other two (Section 4.2) are used to endow the robot with the ability to convey the spontaneous dimension of communication, with one of them providing a more general control over the entire robot’s expressiveness, while the other allows for a more in-depth control, managing specific parameters for each interface. In this work, we focus specifically on the robot’s affective state (i.e., its mood and emotions).

### 4.1. Enhancing the Symbolic Communication through Dynamic Adaptation of Expressions

Giving social robots the ability to adapt their expressiveness to the context of the interaction while communicating with users can help to make these interactions more natural for the users, enhancing the symbolic aspect of communication. For context, we refer to those factors that are external to the robot that play a role in the interaction. This can also help make the communication with the robot more diverse and less repetitive. To this end, we have implemented a modulation strategy that is known as dynamic reconfiguration, which allows developers to modify the appearance of an expression dynamically by changing one or more actions in it. For example, the robot can decide to use a generic *greeting* expression, where it waves its arm and says, *“Hello, how are you?”*, but changing it so that it uses a new utterance. This strategy can also be used to adapt the predefined expressions designed for the robot to the context of the interaction. Continuing the previous example, the generic *greeting* expression can be modified dynamically, so that it references the time of the day (i.e., saying *“good morning”* or *“good afternoon”*, instead of *“hello”*) or the identity of the user.

The robot’s applications can use this modulation strategy by including a list of the actions that have to be adapted and their new configurations in the request that the Expression Scheduler receives for executing a new gesture. These actions will replace those that are predefined in the gesture template. When using this strategy, developers of applications need to know the names of every action in a gesture to be able to indicate which one should be altered. To simplify this process, we used standard names for actions so that they do not change between gestures. It is important to mention that this modulation technique is used to modify existing actions, and does not add new ones or remove actions from the gesture. For example, a gesture that raises and lowers the left arm can be modified so the final positions of the movement are different, but cannot be modified so the gesture also moves the head.

While the addition of the aforementioned modulation strategy allows developers to adapt the robot’s expressiveness to the context of the interaction, it has the drawback of requiring a manual design of the new utterances. The option of using keywords to replace them with information from the context also presents shortcomings, as the structure of the verbal messages itself does not change. While the former can be tedious, the latter might still result in repetitive interactions. In order to correct this, our expressiveness architecture has two methods integrated [15] for automatically paraphrasing the robot’s speech. While one of them seeks only to add variability to the dialogue without changing the message, the other also adapts the way in which the robot phrases its utterances to the profile of the user with whom it is interacting.

For the first paraphrase module, we explored the use of transformers for re-writing the robot’s speech. Because our robotic platforms were designed for interacting with Spanish users in mind, we initially considered two possibilities: (i) fine-tuning a paraphrase model directly in Spanish or (ii) using a model trained in English, and adding translation steps before and after. The advantage of the first approach is a reduction in the time required for obtaining the paraphrased text, while the second approach takes advantage of the considerably larger body of work in NLP and *Large Language Models (LLMs)* in English. After comparing both solutions, our results showed that converting the text to English and paraphrasing it in that language obtained better results [15]. The transformer models that our paraphrase module is able to use are PMO-T5, Parrot, PEGASUS, and GPT-3. Regarding the translation of utterances from Spanish to English and back, we integrated three options: the Google Translate (https://cloud.google.com/translate/, last accessed on 25 May 2024), DeepL (https://www.deepl.com, last accessed on 25 May 2024), and Argos translators (https://pypi.org/project/argostranslate/, last accessed on 25 May 2024).

The previous approach allows us to enhance the interactions with our robot by introducing variability in its dialogues, which in turn makes them less repetitive and more natural. However, this method does not take into account any contextual factors that might affect communication in humans, in particular the profile of the person the robot is interacting with. While for short or menial conversations, this might not be an issue, when communicating complex topics, it could be beneficial to adapt the robot’s speech to the user in order to enhance his/her experience. For this, we have developed a second paraphrase module that exploits the capabilities of transformer models, and in particular *LLMs*, for adapting utterances to the user [15]. In particular, our approach uses zero-shot learning with GPT-4, building a prompt that tells the model that a user with a series of identifying features has asked what the robot’s speech means. This leads the model to rephrasing the text in a way that is easier to understand for a person with that particular profile. Currently, the user features that our module considers are the user’s name and his/her age. While the structure of the prompt is always kept constant, the module modifies it to add the information about the user, and concatenates the text that has to be paraphrased. The experimental results regarding the performance of both paraphrase approaches can be found in [15].

When it comes to endowing our robots with multimodal communication, the Expression Manager gives developers multiple solutions for this. However, all of them require that the developers manually define the verbal and non-verbal components of the robot’s expressiveness, either by defining individual actions for each interface (i.e., specify the trajectories for the motors, the expression for the eyes, and the sentence that has to be uttered) or by selecting one of the robot’s predefined expressions (i.e., asking the Expression Manager to perform a greeting gesture alongside an utterance). This can be time consuming, particularly in applications where developers only care about the verbal messages that the robot conveys, but still want to add non-verbal expressiveness that enhances the verbal communication. While the Liveliness module in our architecture generates random non-verbal actions aimed at giving the robot a lively appearance, these actions are generic, and do not complement the verbal messages in any way. To correct this issue, we have integrated in our architecture a module that automatically selects from our gesture library the expressions that should accompany the robot’s utterances [16]. We have formulated the problem of gesture prediction as a token-classification task, where the robot’s speech is divided into a sequence of tokens. Then, each token in the sequence is labelled with the type of gesture that should accompany it. We fine-tuned three transformer models: BERT, DistilBERT, and RoBERTa. All three models have been pre-trained on language modelling. During the fine-tuning process, we added a new token classification head (a linear layer connected to the output of the hidden states) to the base models. The dataset used for fine-tuning the models contained 21 gesture types. Examples of this include ***greet*** (gestures where the robot greets the user), ***self*** (gestures where the robot points at itself), or ***emphatic*** (gestures where the robot makes wide and aggressive motions, in order to emphasise the point being made with the verbal message). First, the gesture-prediction module labels the robot’s utterance with the type of gestures that should accompany it (a utterance can be accompanied by none, one, or multiple gestures). Then, the module selects from the gesture library one expression for each different gesture type, ensuring that the duration of the gestures is less than the duration of the speech chunks they are attached to. If, for a given label, multiple expressions fit this time constraint, one is selected randomly. Finally, the prediction module returns the utterance, and a list of the gestures that have to be performed alongside it, with the moment in time (measured in seconds) in which each expression has to start. These time points are measured from the moment the speech starts to be uttered, and computed based on the length the text.

Both paraphrasing strategies and the gesture prediction system have been integrated in our software architecture as independent modules that communicate directly with the Expression Manager. In the work where the paraphrasing methods were originally presented [15], they were integrated as part of the Emotional Text-To-Speech (ETTS) Player. This module of the Expression Manager sends the utterances that have to be spoken to the ETTS, and ensures that the message is properly conveyed. However, in order to integrate both paraphrasing approaches alongside the gesture-prediction module, we modified this solution, giving control over the communication with these modules to the Expression Scheduler. This was performed because the paraphrasing has to be performed before the prediction of gestures, as the timing of these expressions depends on the speech’s transcription. During startup, the Expression Manager loads a configuration file that, among other features, indicates if any of the paraphrasing methods and the gesture-prediction module are available, and if they are running locally or externally. All three modules use Deep Learning models, which have a high requirement of system resources when running inferences. This can lead to long inference times, which can affect the quality of the interactions if the models are deployed on the robot, as its hardware was not designed with machine learning in mind. In order to solve this issue, we prepared all three modules to be run either on the robot or on an external server designed specifically for running Deep Learning models. This server is equipped with an Intel Core i9-10900K CPU running at 3.7 GHz, two NVIDIA GeForce RTX 3090 GPUs, and 64 GB of RAM. The robot communicates with this server through a socket-based connection. Whenever the Expression Scheduler receives a request for executing a new gesture, it first checks which interfaces will be used. If the requested expression contains a verbal message, the Scheduler checks if any of the paraphrasing methods is available. If both are available, the method that adapts the text to the user will always be selected if the robot has a profile for the user it is interacting with. If the robot is interacting with a new user for whom we do not have a profile, then the basic paraphrasing module is selected. The Scheduler sends the utterance to the paraphrasing method selected and waits for the result to be returned. After paraphrasing the text, if the requested expression only contains the verbal message, this is sent to the gesture-prediction module. Finally, once the prediction module returns the utterance and the gestures that have to be performed, the Scheduler continues with the process of executing the expression described in Section 3, as would be performed for any regular gesture.

### 4.2. Conveying the Spontaneous Dimension of Communication

**Figure 4:**
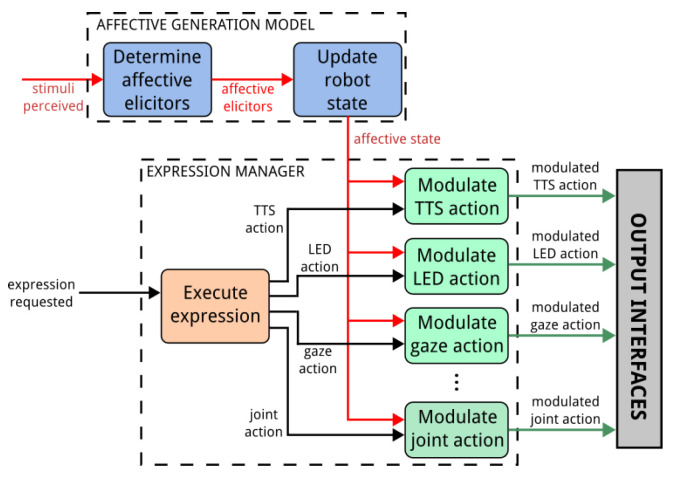
Process followed for expressing Mini’s internal state. Actions in blue blocks are performed in the affect-generation module; actions in orange blocks are performed in the Expression Executor; actions in green blocks are performed in the Interface Players.

When considering how to convey the spontaneous component of communication (i.e., the internal state of the robot), we decided to focus only on its affective state, that is displaying a combination of mood and emotions. The affect-generation model that has been integrated in Mini was first presented in [47]. As mentioned in Section 1, we consider emotions to be short-lived events with high intensity, while moods are long-lasting and have a lower intensity. Figure 4 shows the process for expressing Mini’s affective state.

In our robot’s architecture, moods and emotions are represented in two separate valence–arousal spaces—both with axes that range from −100 to 100 units. The robot’s affective state is then defined as a time-dependent state that results from blending the robot’s dominant emotion (i.e., the emotion with the highest intensity) and mood. While the robot will always have an active mood, emotions can be triggered as a response to stimuli perceived from the environment. For example, hitting the robot will result in an increase in arousal and a decrease in valence. Mini is able to experience four out of Ekman’s six basic emotions (i.e., joy, sadness, anger, and surprise), with different levels of intensity (a continuous, 0 to 100 value). More than one of these emotions can be triggered at any given time, with different intensity levels. Moods, on the other hand, are discrete, and only one can be experienced at any given time. The ones considered are happy, anxious, bored, and relaxed.

When designing the expressiveness component of the affect expression, it is important that this display of affect is properly blended with any display of task-related information. When looking for a solution to this problem, our focus was to free expression developers from the need to consider affect when creating the robot’s gestures, but to still give them the tools to handcraft the modulation that a particular expression requires if they need to. This is the reason why we decided to develop two different modulation strategies for fusing the symbolic and spontaneous aspects of communication:

1. Parameter-based approach: This technique allows us to modify an individual expression in a speed–amplitude space, making it faster or slower, and/or more or less intense.
2. Profile-based approach: This strategy uses profiles to modify specific characteristics of the robot expressiveness depending on the state that has to be conveyed (e.g., happiness may be connected to an increase in the voice’s pitch).

The *parameter-based* modulation strategy alters the global appearance of the robot’s expressions in two dimensions: *speed* and *amplitude*. *Speed* is tied to the temporal aspects of the expressions, and altering it translates into different effects depending on the interface. For example, increasing the *speed* of an expression will increase the articulation rate of the robot’s voice, the speed of the motions, or the speed at which the robot’s eyes blink, among others. *Amplitude* alters the intensity of the gesture. This includes features such as the amplitude of the motions, the pitch of the voice, or the brightness of the robot’s LEDs. The effect that each parameter has on the different interfaces can be seen in Table 1. The applications of the robot are the ones that define the value for the speed and amplitude parameters when requesting expressions to the Expression Manager. It is important to mention that these modifications will be applied to all of the features and cannot be decoupled. The idea is to change the general way in which the user perceives the robot’s expressions, making them look *faster* or *slower* or more or less *intense*, but without focusing on any communicative interface in particular. To simplify the use of this modulation method by the application developers, we decided to limit the possible values for the speed and amplitude parameters to seven options, as follows: big decrease, medium decrease, small decrease, normal, small increase, medium increase, and big increase. The modulation is then applied for each parameter in Table 1 according to Equation (1):

(1) $$V_{f}=V_{i}+s*\left(V_{l}-V_{i}\right)$$

In this equation, $V_{f}$ is the value after modulation, $V_{i}$ is the value that is predefined in the gesture, $V_{l}$ is the limit value (maximum or minimum, depending on if the modulation is an increase or decrease) that the particular feature can take, and *s* is the size of the modulation. The limit values for the parameters in Table 1 were defined experimentally, and represent a threshold beyond which an action might not be perceived as being natural. For example, while the robot’s voice could have a higher pitch than the one defined as the top limit, the resulting voice might no longer be perceived as human-like and, thus, could affect the quality of the interaction. Regarding the size of the modulation, *s* will take the value of 0.33 for small modulations, 0.66 for medium modulations, and 1 for big modulations, which would in turn set the parameter to its limit value.

Finally, the *profile-based* modulation strategy uses a series of modulation profiles to define how each of the possible internal states that the robot might experience changes how its expressions are performed. The modulation profiles are handcrafted by roboticists, and each profile is tied to a particular type of internal state. For example, one modulation profile can be used to adapt the robot’s expressiveness to the *emotion* that the robot is experiencing, and this profile would contain different modulations for each possible *emotion* considered. If the internal state of the robot changes, then the expressiveness architecture checks the profiles, retrieves the configuration connected to the new state, and applies it to any requested expression. It is important to mention that, when developing this modulation approach, we assume that the coherence between the task-related information defined by the developers of the robot’s applications and the robot’s internal state is being guaranteed by the rest of the software architecture. For example, we assume that if the task being performed requires that the robot conveys a sentence with a strong positive sentiment (e.g., cheering the user after they perform well during a game), the module in charge of defining the robot’s internal state will take this into account and ensure that the robot’s state does not contradict the sentiment of this utterance.

As mentioned earlier, having developers create the modulation profiles gives them a deeper control of how the robot’s expressiveness looks under every considered internal state, but at the same time, can be time consuming if the number of internal states is too high. In our robots, we considered two types of internal states, *emotions* and *moods*, and defined four possible states for each of them. This resulted in nine possible internal states (i.e., four emotions, four moods, and the *neutral* state). With this number of internal states, we considered that the benefits of being able to tailor the modulation through the manual design of the profiles outweigh the time required for creating them. Table 2 shows an example of the differences between the configurations for the *neutral* and *sad* emotions.

The values that the parameters of each interface should take depending on the robot’s internal state are defined in the profiles as a percentage of the range of possible values that each particular parameter can take. For example, if the prosody rate can vary from 0.65 to 0.85 (these limit values are the same ones used for the parameter-based modulation approach), then 50% would translate to a value of 0.75, while 100% would translate to a value of 0.85. Developers can store configurations for multiple states of the robot in each profile. During the initialisation of the system, each of the Interface Players loads from the profiles the information related to that particular interface. It then sets the configuration defined as *neutral* as the starting one. Whenever a new goal is sent to a Player, the default value assigned to any feature of the interface not defined in the expression will be modulated using the configuration from the profile. For example, if an expression that uses the robot’s voice requests using a specific pitch value, it will be used regardless of the robot’s state. However, if no value is defined for the pitch, the ETTS Player chooses it using the active modulation profile. If the Player receives a message notifying about a change in the robot’s state (i.e., the robot goes from a neutral mood to being happy), then the configuration related to the new state is set as active.

There are three main differences between both techniques: (i) the *parameter-based* technique allows us to modify individual expressions without affecting the rest, while the *profile-based* one alters all the expressions; (ii) the *parameter-based* affects all the interfaces of the robot at once, while the *profile-based* one allows defining individual effects for each interface; (iii) the *parameter-based* strategy is usually controlled by the robot applications, while the *profile-based* strategy is internal and independent of the task at hand. Thus, when developing applications for the robot, roboticists can decide which strategy better suits their needs at any moment.

## 5. Evaluation

As stated in the Introduction, we sought to test if a proper merging of the two dimensions of communication described by Buck and VanLear during human–robot interactions can significantly enhance the way in which users perceive a social robot. For this, we conducted two user studies. Both experiments were designed using Mini as the robotic platform. Although this robot was designed for assisting adults who are older, we developed the Expression Manager for interacting with a broader spectrum of population. Thus, for the experiments, we contacted participants of all ages, not only people who are elderly. In the first experiment, we studied the effect that the addition of the spontaneous component of communication has on how people perceive a social robot. For this, we compared a neutral robot and a robot that can express affective states using the profile-based modulation strategy. The second experiment tests how a proper fusion of both the symbolic and spontaneous dimensions of communication can alter the perception that users have of the robot, in contrast to a robot that can use both components separately. The comparison was between a robot that uses gestures without any modulation against a robot that adapts its expressiveness to the context of the interaction and its internal state, using the dynamic reconfiguration and parameter-based modulation strategies. Following other studies in robotics [48,49,50], we decided to use questionnaires to conduct these studies. Both evaluations were conducted using videos, instead of in-person evaluations. This has the advantage of simplifying the process of reaching participants, which in turn allows for a larger population with a wider range of profiles (ages, education level, etc.), and also ensures that all participants have the exact same experience and perceive the experimental conditions in the same way [51]. These two experiments also serve as a proof-of-concept of our system being used in a real robot and in real-world applications.

### 5.1. Evaluation of the Response Time and Resource Consumption

When developing a software module that will play a role during interactions between a robot and a user, there is a series of considerations that need to be kept in mind. First, robotic platforms have a limited amount of computational power and memory, and these resources have to be shared among all the modules in the software architecture. Because of this, we need to ensure that the proposed expressiveness architecture has an acceptable performance, as regards CPU and RAM usage. Also, interactions between humans have temporal constraints that have to be met for the interaction to be natural and for ensuring the coherence of the information shared. The *“two-second” rule* [52] states that responses from a computer stop being natural after two seconds. While this rule has been validated for robotics (see [53]), other authors have found that users preferred response times under 1 s [54]. Time constraints are different for reactive behaviours (e.g., being startled when frightened). According to research [55], this reaction time is around 200–250 ms for responding to a sudden impulse and around 380 ms for identifying if a particular stimulus requires a response. Thus, to evaluate if our approach is usable in real scenarios, we will compare the reaction time of the system to all three thresholds (0.25, 0.38, and 1 s)

All of the measurements were taken in Mini, which is equipped with an Intel i5-3550, with four cores running at 3.3 GHz, and 16 GB of RAM. The operating system running in the robot is Ubuntu 16.04. To evaluate the response time of our system, we requested the execution of six different expressions. The first five were expressions that had a single unimodal action (i.e., uttering a sentence, sending a single position to a joint, changing the state of one of the robot’s LED, etc.). This represents the most favourable situation for the Expression Manager, as they are the most basic expressions that could be requested. For the last case, we selected an expression where the robot waved its right arm while shaking its head from side to side. In total, the expression included 20 actions (10 different points for each of the two interfaces). This is a bad case scenario for the Expression Manager, as few expressions include such a high number of independent actions (each represented as a separate state in the state machine). The reason behind this is that this expression was one of the first ones created, and was poorly optimised (we can achieve the same result with an expression with only two states, one for sending the full 10-point trajectory to the neck motor and another for sending the full 10-point trajectory to the arm). For evaluating the use of resources, we measured the usage of the CPU and RAM when the system receives a request for executing a gesture that makes use of all the robot’s interfaces at the same time (sends one action to each actuator). This would be the worst case scenario, where all the modules of the expressiveness architecture are working at the same time. We ran each test 10 times and computed the average value.

**Figure 5:**
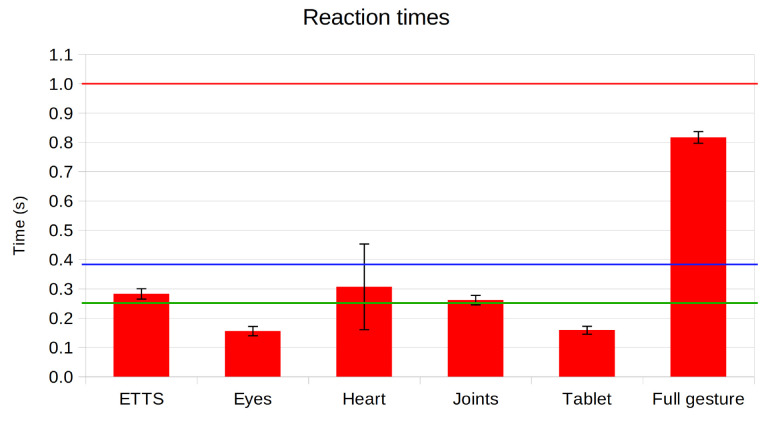
Response time for the Expression Manager, understood as the time that passes between the moment a gesture request is sent until the first action is sent to the output interface. Bars represent the average value; whiskers represent the standard deviation; the green line represents the threshold for responding to a stimulus; the blue line is the threshold for identifying if a stimulus requires a response; the red line is the threshold we have defined for conscious interactions. Bars with the name of an interface correspond to expressions with a single action, while Full gesture corresponds to a multimodal gesture that performs multiple actions.

The results for the resource usage test showed that the system consumed around 2.2–3.3% of the available RAM and around 42.2% of one of the cores’ power. While the RAM usage is acceptable, there is a need to optimise the use of the CPU. All of the expressions performing a single action met the time constraints identified above, while the multimodal gesture only met the 1 s threshold for naturalness (0.82 s). These results, which can be seen in Figure 5, show that the proposed system can be used for human–robot interactions, although it can have problems managing complex reactive behaviours. This also indicates that it could be beneficial to improve the efficiency of the Expression Manager when performing complex expressions.

### 5.2. First User Evaluation: Conveying the Spontaneous Aspect of Communication

In this first evaluation, we tested how the use of the profile-based modulation for conveying the spontaneous dimension of communication can improve the perception that users have of a social robot. We decided to use the expression of affective states as an example of inner states that can be conveyed using these profiles. In this experiment, Mini is able to display emotions and moods in three different ways. First, it can use handcrafted expressions to convey punctual emotions to react to certain stimuli. For example, Mini performs an angry expression to complain if the user hits it. Second, all of the expressions are modulated to convey the mood that Mini is in at any moment. Finally, if an emotion arises, then Mini’s expressions start to be modulated to convey the elicited emotion with different intensity levels. The emotion and its effect on Mini’s expressiveness decay with time, and the expressions go back to convey only the mood once the elicited emotion disappears.

#### 5.2.1. Experiment Definition

**Figure 6:**
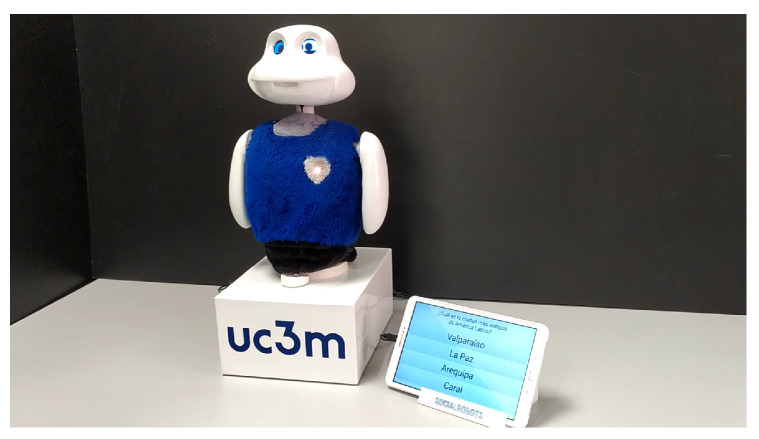
Mini during the quiz game conducted as part of the evaluation. The robot’s tablet shows the question the robot has asked and the four possible answers (all of them appear in Spanish).

**Figure 7:**
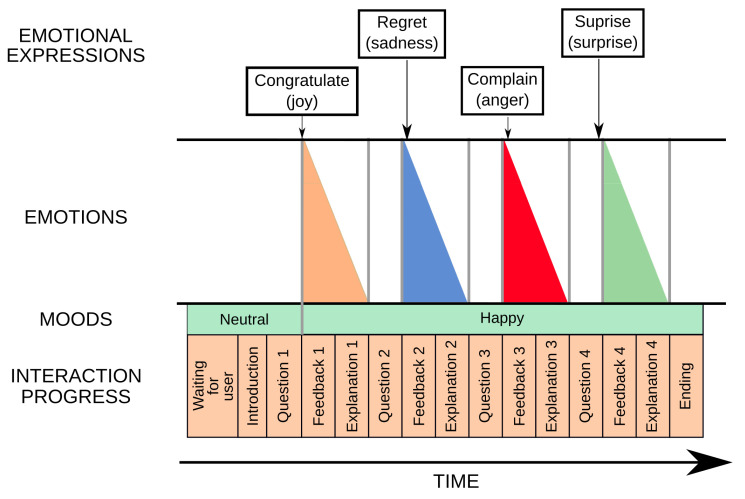
Evolution of the affective state expression during the experiment.

In this experiment, the participants observed a video-recorded interaction between Mini and a user where they played one of the games that our robot is able to play: a quiz game. In this game, the robot asks a series of questions about a specific topic of the user, who has to answer by selecting the correct option among those displayed on the touch screen. A caption of the interaction can be seen in Figure 6. Mini starts in an idle state, waiting for the user to press a button on its touch screen. Once the user starts the interaction, Mini wakes up and introduces itself and the rules of the game. The user then selects the category of questions for the game (in this interaction, the selected topic was History), and the game begins. Mini asks four questions and gives the user multiple options. After the user answers each question, Mini tells him/her if his/her answer was correct or not, gives more information about the correct answer, and then performs a short interaction with the user. During the game, the user correctly answers the first question, which elicits the *happy* emotion. This leads to Mini performing the *congratulate* expression (as the emotion was elicited by the correct answer). Then, he/she gives the wrong answer to the second question, which elicits the *sad* emotion and the execution of the *regret* expression. After the third answer, the user hits Mini because he/she considers that the robot is cheating. This triggers the *angry* emotion, which leads to Mini performing an *angry* expression to reprehend the user. Finally, after giving a correct answer to the last question, the user became excited about having answered correctly and patted Mini on the belly, which elicited the *surprise* emotion. The robot reacted to this by performing the *surprise* expression. Figure 7 shows how the emotions and moods evolved during the quiz game, as well as the moments when emotional expressions are requested. The entire interaction can be seen in a YouTube playlist (https://youtube.com/playlist?list=PLxGCA0SJbjmlwUk1LSsWZUxP-9bnUw7aa, last accessed on 25 May 2024).

#### 5.2.2. Conditions

For this experiment, we developed two conditions. Under the *neutral condition*, the robot does not convey affective states, and is only able to express the symbolic component of communication. To ensure that the interactions for both conditions are the same, the gestures for displaying punctual expressions of emotion have been replaced with neutral versions (i.e., no variation of the pitch, prosody rate, or volume). Under the *expressive condition*, the robot conveys both components of communication, and uses expressions for conveying punctual emotions with all the interfaces and the emotional speech (i.e., the prosody rate, pitch, and volume of the voice are altered to convey a particular emotion). The decay rate of the emotions is adjusted so they would last for the whole explanation after each question. Finally, Mini’s mood goes from neutral to happy after the user gives the correct answer to the first question. Mini conveys the triggered emotion during the explanations of the answers, and goes back to conveying only mood during the brief interactions with the user between questions.

#### 5.2.3. Questionnaire

The participants in this experiment evaluated the robot by completing an online questionnaire. First, they had to complete a series of demographic questions about their age, their education level, the familiarity they had with technology and robotics, and their disposition to interact with and own a robot. In this section of the questionnaire, we included a control question to ensure that participants did not complete the questionnaire twice by accident. Next, participants were presented with a video of the robot and were asked to watch it entirely. After watching the video, they had to give their perception of the robot. We used the RoSAS questionnaire [56] to perform this evaluation. According to this test, the robot will be evaluated as regards the following three dimensions: *warmth*, *competence*, and *discomfort*. Each item in the RoSAS scale was rated using a 9-point Likert scale. We added a second control question in which the participants had to given an answer to how many robots appeared in the video. We added this question to confirm that the participant was paying attention to the video. Those participants who gave the wrong number or robots were discarded and their responses were not used. After evaluating the perception of the robot with the RoSAS questionnaire, we added two questions to specifically measure how the participants perceived the robot’s mood. First, participants were asked if they perceived any mood in the robot during the interaction. If they selected the option “Yes”, then they were presented with a second question that asked them to write in a text box which mood they perceived. Finally, the participants were allowed to post comments and suggestions. We developed two versions of this questionnaire, one for each condition. We assigned participants randomly to each condition to balance the number of answers.

#### 5.2.4. Hypotheses

For this experiment, we considered the following hypotheses:

- H1.1: The participants’ perception of Mini will be affected by the modulation of the robot’s expressiveness.
- H1.2: Mini will be perceived as being *warmer* under the *expressive* condition, when compared with the *neutral* condition.
- H1.3: Mini will be rated higher in the *discomfort* dimension under the *neutral* condition, when compared with the *expressive* condition.
- H1.4: Mini’s rating for *competence* will not show a significant difference between both conditions.

#### 5.2.5. Participants

We recruited a total of 83 persons through email and messaging applications, 38 for the *neutral* condition and 45 for the *expressive* condition. In total, 36 of the participants identified themselves as male, while the remaining 47 identified themselves as female. The average age of the participants was 36.02 years old, ranging from 16 to 63. There were 53 participants who had a college education (Bachelor’s or Master’s degrees, or a Ph.D.), and 89% of the participants showed at least a medium level of familiarity with technology, while this percentage fell to a 43% for familiarity with robotics. Regarding their willingness to interact with and own a robot, the percentages of users with at least a medium level of interest were 85% and 78%, respectively.

#### 5.2.6. Validation of the Emotional States Conveyed by Mini

**Figure 8:**
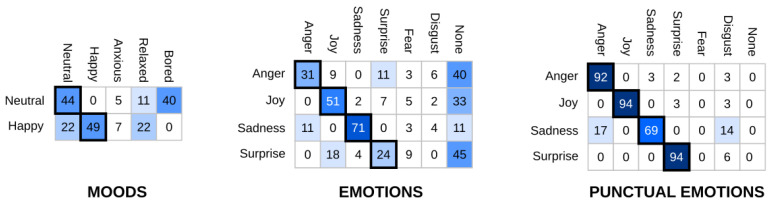
Confusion matrices representing the results of the affective state-recognition evaluation. Rows in the matrices represent the affective state the robot was expressing, while columns represent the options selected by the participants (as a percentage). Cases where participants selected the correct affective state have been highlighted with a thick black border. The intensity of the colour is directly tied to the percentage of participants selecting each option.

Before evaluating the effect that the addition of the modulation strategies has on how the robot is perceived by the participants, we need to ensure that the modulation itself is being correctly perceived, that is that the affective states that the robot is supposedly conveying are identified as such by the users (i.e., the “happy” expression is perceived as happy, or the robot is seen as being sad when the “sadness” emotion is triggered, etc.). To evaluate this, we recorded videos of the robot displaying the different moods and emotions used in the interaction described earlier, as well as the emotional expressions. In total, 55 participants watched all of the videos and selected the mood/emotion that they believed the robot was trying to convey. The participants were presented with a list of possible options and were asked to select one of them. As can be seen in Figure 8, the results of this pre-evaluation showed that the mood was the hardest to perceive, although the most selected option was the correct one. In particular, 44% and 49% of participants correctly identified the “neutral” and “happy” moods, respectively. Regarding the recognition of emotions, “surprise” and“anger” were the only affective states that were not properly recognised by the participants, as 40% and 45% of them, respectively, considered that the robot was not expressing any emotion, as opposed to 31% who correctly identified “anger” and 24% who correctly identified “surprise”. For the other emotions, “joy” was correctly identified by 51% of the participants and “sadness” by 71% of them. Finally, the expressions used for conveying punctual displays of emotion were easier to recognise. The recognition rate was 92% for “anger”, 94% for “joy”, 69% for “sadness”, and 94% for “surprise”. These results led us to consider that the modulation profiles and the expressions for conveying punctual displays of emotion were correctly designed. Once we were sure that the robot would be able to correctly express its affective state, we moved on to organising the study.

#### 5.2.7. Results

**Figure 9:**
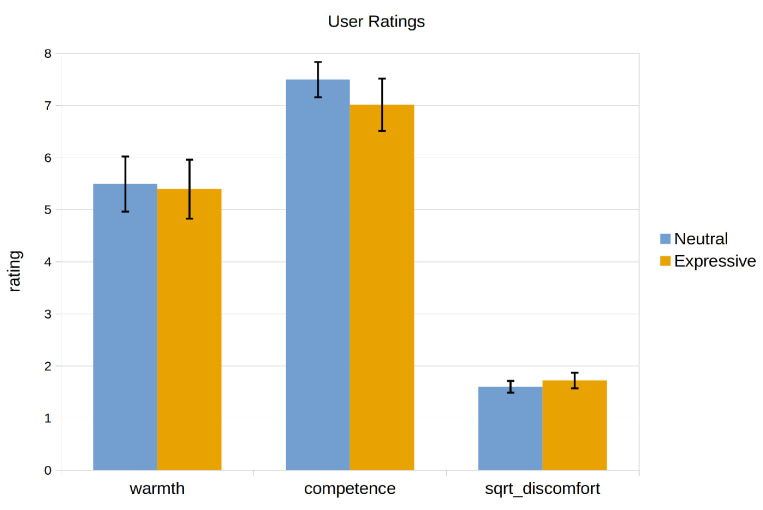
Average value for the ratings computed for each of the dimensions on the RoSAS questionnaire (warmth, competence, discomfort) for both conditions. Bars represent the average rating, while whiskers represent the 95% confidence intervals.

We started by searching for duplicate answers and participants that provided the wrong answer to the control question in the questionnaire. No such cases were identified, and thus, the answers provided by all 83 participants were kept. We then computed the dimensions for the RoSAS questionnaire, and performed normality tests for each dimension and condition using the Shapiro–Wilk test. The results of the test showed that only the ratings for *warmth* followed a normal distribution ($W_{neutral}\left(38\right)=0.977,p=0.599$, $W_{expressive}\left(45\right)=0.982,p=0.707$). To achieve normality, we performed a square root transformation and repeated the Shapiro–Wilk tests. After the transformation, the ratings for *sqrt_discomfort* now also followed a normal distribution ($W_{neutral}\left(38\right)=0.967,p=0.317$, $W_{expressive}\left(45\right)=0.956,p=0.084$). For the last dimension (*competence*), we analysed the Q-Q plots and observed that the data aligned with the diagonal line representing a normal distribution. Thus, we considered that normality could also be assumed for the *competence* dimension. We then performed a descriptive statistics analysis for the ratings of *warmth*, *competence*, and *sqrt_discomfort*. This analysis showed that the ratings for *warmth* and *competence* were higher under the *neutral* condition, while the rating of *sqrt_discomfort* was higher under the *expressive* condition. The average ratings for each of the dimensions of the RoSAS questionnaire can be seen in Figure 9. To evaluate whether or not these differences are significant, we conducted Independent Samples T-Tests, with Levene’s tests to ensure the homogeneity of variances. The results showed that none of the differences observed were significant.

### 5.3. Second User Evaluation: Dynamic Adaptation of the Symbolic and Spontaneous Dimensions of Communication

With this second evaluation, we wanted to test if adapting the symbolic and spontaneous communication to the context of the interaction enhances the perception that users have of it, when compared with a robot that uses only predefined symbolic messages. For this second evaluation, we modulated the robot’s expressiveness using the *dynamic reconfiguration* and *parameter-based* modulation strategies.

#### 5.3.1. Experiment Definition

We used a scenario similar to the one that was developed for the first experiment (i.e., the robot playing a game with a user). In this game, Mini displays on the touch screen pictures of landmarks from all around the world, and the user is supposed to answer in which city that landmark is located. The game involved 4 questions. In the interaction that we recorded, the robot started in a state that simulated being asleep. The user is sitting in front of the robot, although not seen in the shot. The user then wakes up the robot by touching it on the shoulder, and Mini performs the *Wake up* gesture. After introducing itself, Mini informs the user that he/she is about to play a game and explains the rules. The user gives the correct answer to the first two questions, and then misses the last two. Mini performs the same *Congratulation* gesture after every correct answer, while performing the same *regret* gesture after every wrong answer. The interaction ends with Mini thanking the user for playing the game, before wishing his/her goodbye. The entire interaction can be seen in a YouTube playlist (https://youtube.com/playlist?list=PLxGCA0SJbjmlYpqjy5ccq4E2zrb_fZH-E, last accessed on 25 May 2024).

#### 5.3.2. Conditions

**Figure 10:**
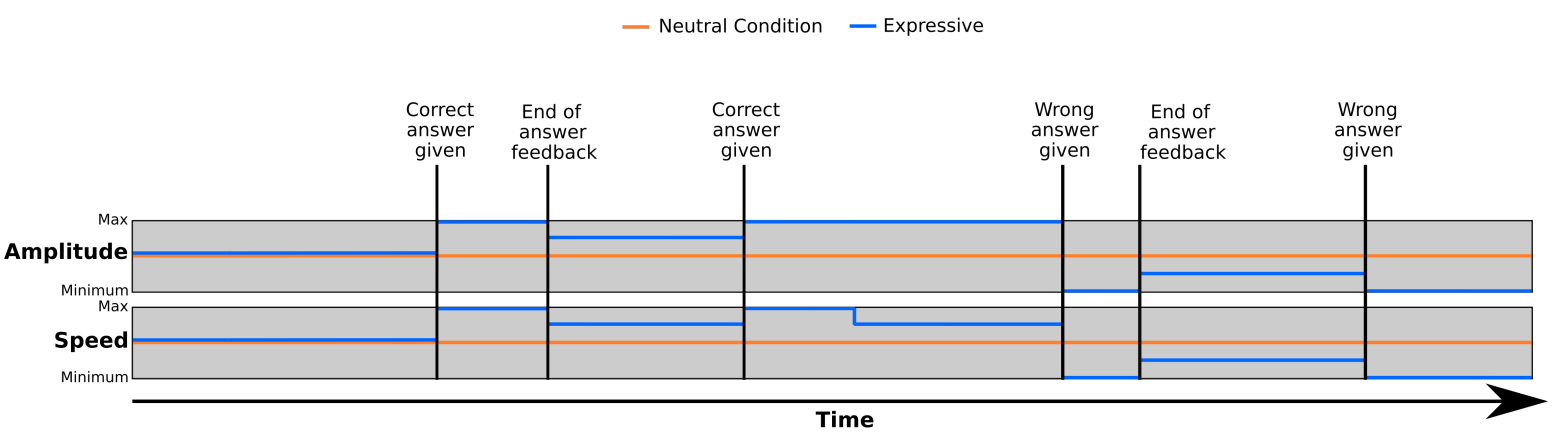
Evolution of the amplitude and speed parameters during the game of guessing the location of famous landmarks.

As in the first study, we developed two conditions. Under the *neutral condition*, Mini repeats the same feedback gestures in questions 1 and 2 (*congratulate*) and in questions 3 and 4 (*regret*). The connector between questions is also the same (*“Let’s move on to the next question”*). Finally, the amplitude and speed modulation parameters were set to *normal* for the whole interaction. This means that all the features in Table 1 remained constant. Under the *expressive condition*, the feedback questions were adapted to the user’s performance during the game through the modulation of the *amplitude* and *speed* parameters, as shown in Figure 10. For the *Congratulation* gesture, the amplitude was set to *High increase* and speed was set to *medium increase*, while both were set to *High decrease* for the *regret* gesture. Both the amplitude and speed increased after each correct answer, and decreased after each wrong one. This sought to convey the impression that the excitement of the robot grows after every correct answer, while the robot becomes sadder with every wrong answer. Finally, the utterance for transitioning between questions is adapted to the context of the interaction. After question 2, the robot makes a reference to the fact that the user has given two correct answers in a row. After question 3, Mini tries to encourage the user to perform better on the next question. Finally, after question 4, Mini references the fact that the user has given two wrong answers in a row.

#### 5.3.3. Questionnaire

For this experiment, we used the same questionnaire that was used in the first evaluation, where participants evaluated how they perceived the robot using the RoSAS questionnaire. Again, two versions of the questionnaire were used, one for each condition, and the participants were randomly assigned to each condition, ensuring a balanced split of the participants between conditions.

#### 5.3.4. Hypotheses

The hypotheses considered for this experiment were the same as those that we evaluated during the first subjective test:

- H2.1: The participants’ perception of Mini will be affected by the modulation of the robot’s expressiveness.
- H2.2: Mini will be perceived as being *warmer* under the *expressive* condition, when compared with the *neutral* condition.
- H2.3: Mini will be rated higher in the *discomfort* dimension under the *neutral* condition, when compared with the *expressive* condition.
- H2.4: Mini’s rating of *competence* will not show a significant difference between both conditions.

#### 5.3.5. Participants

We contacted the participants for the study through email and messaging applications. In the end, 69 participants took part in the experiment, with an even split between conditions. Regarding the gender distribution, 34 out of the 67 participants identified themselves as male, while the rest marked the option *female*. The average age of the participants was 33.94 years old, ranging from 19 to 67. Meanwhile, 74% of the participants had a college education (either a Bachelor’s or a Master’s degree), 87% reported at least a medium level of familiarity with technology, while almost 80% of them reported a medium or lower familiarity with robotics. Finally, 81% of the participants had a medium or higher interest in interacting with, and owning, a robot.

#### 5.3.6. Results

**Figure 11:**
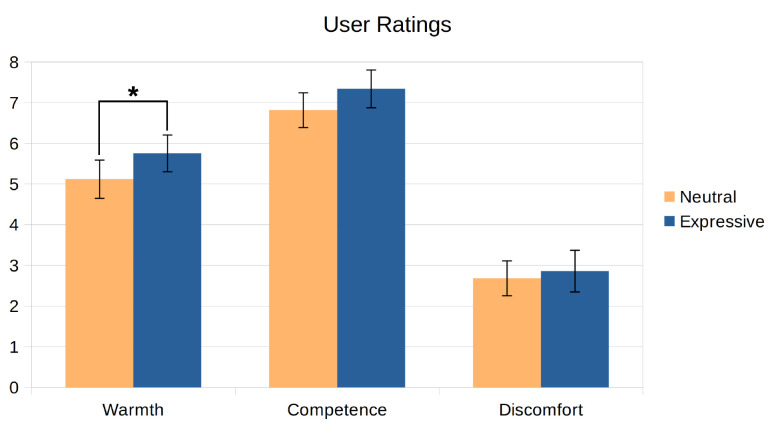
Average value for the ratings computed for each of the dimensions on the RoSAS questionnaire (warmth, competence, discomfort) for both conditions. Bars represent the average rating, while whiskers represent the 95% confidence intervals. The bars connected with an asterisk are those for which significant differences were observed.

**Figure 12:**
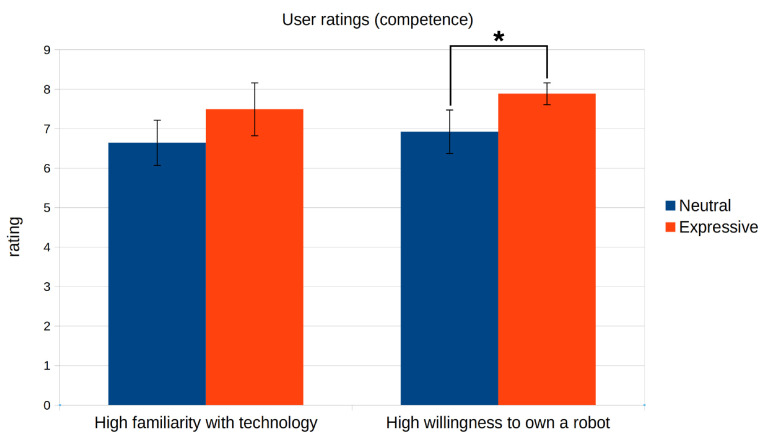
Average value for the competence rating when considering only users that reported either a mid-high or high interest in owning a robot and users that reported either a mid-high or high familiarity with technology. Bars represent the average rating, while whiskers represent the 95% confidence intervals. The bars connected with an asterisk are those for which significant differences were observed.

Once the pre-processing stage was completed, we performed a descriptive statistics analysis for all three variables (i.e., *warmth*, *competence*, and *sqrt_discomfort*). As shown in Figure 11, all three dimensions presented higher ratings under the *expressive* condition, although the difference was smaller for the *sqrt_discomfort*. To evaluate if these differences were significant, we performed Independent Samples T-Tests with Levene’s tests to ensure the homogeneity of variances. The results showed that there was a significant difference for the ratings of *warmth* (t(65)=−2.173,p=0.033), while no significant differences were observed for the other two dimensions of the scale. We also wanted to analyse if the profile of the participants could affect the way in which they were perceiving the robot. For this, we performed an analysis of covariance (ANCOVA) using the variables *familiarity with technology*, *familiarity with robotics*, *willingness to interact with a robot*, and *willingness to own a robot* as co-variables. When controlling for these co-variables, we observed marginal differences for the rating of *competence* when taking into account the familiarity with robotics ($F=3.845,p=0.067,\eta^{2}=0.051$). Finally, to know more about these differences in the ratings of *competence*, we divided the data based on the responses given by the participants to the questions used as co-variables and repeated the Independent Sample T-Tests. We found significant differences in the ratings of *competence* for users that reported either a mid-high or high interest in owning a robot (t(29)=−3.122,p=0.004) and marginal differences in the ratings of *competence* for users that reported either a mid-high or high familiarity with technology. These results are shown in Figure 12.

### 5.4. Discussion

Overall, the results obtained from the subjective evaluations suggest that the modulation strategies integrated in our expressiveness architecture can improve the perception that users have of the robot, based on the results for the second evaluation. Also, the results suggest that these strategies can also be used to convey internal states in a recognisable way, based on the results obtained from the pre-evaluation conducted as a preparation for the first study. First of all, the pre-evaluation conducted showed that participants were able to recognise the moods and emotions being displayed above chance level. In the case of moods, participants were given five options to choose from, which sets the chance level at 20%. Both moods used in the experiment were recognised by more than 40% of participants. For the identification of both emotions and punctual displays of emotion participants were allowed to choose from a list with seven options, which translates into a 14% chance of randomly selecting the correct option. In the worst observed case (“surprise”), 24% of participants were able to correctly recognise the emotion the robot was conveying. This means that the proposed profile-based modulation strategy can be used to convey recognisable affective states without hindering the expressiveness of the robot. In the first evaluation (Section 5.2), none of the differences observed between conditions proved to be significant, which means that hypothesis *H1.1* was not validated. The results obtained validated hypothesis *H1.4* because there were no differences for how competent the robot appeared to be under the *expressive* and *neutral* conditions. The lack of any significant differences in the ratings of *warmth* and *discomfort* mean that hypotheses *H1.2* and *H1.3*, respectively, were not validated.

Other authors have also evaluated how users perceived emotions conveyed by a social robot, although there is no consensus when trying to identify which emotion is the hardest to identify. Among the works reviewed in Section 2, [42] observed that positive emotions were easier to identify than negative emotions. In contrast, [19] reported that anger was more difficult to recognise than happiness or sadness. Our results are in line with those presented by [57], who also found that surprise is the hardest emotion to identify.

We considered a series of explanations for the lack of significant differences between conditions in the study. One of the possible reasons is the fact that the robot was specifically designed to be warm and friendly—both in its external aspect, as well as the configuration of the interfaces that we defined as being neutral. This could mean that the robot was not actually being perceived as not conveying any affective state under the *neutral* condition. Another possible explanation is that the duration of the scenario chosen for the experiment is not long enough to appreciate a correct evolution of the emotions and moods; for example, Mini went through four emotions and two moods in the span of minutes, which is not a very realistic situation. A third possible reason, which actually ties into the previous one, is that we designed the configurations for the affective states with the goal that they were clearly distinguishable in videos. For this, we sought to create representations of those states with a high intensity. While this could have helped participants identify each particular emotion and mood, it could have also resulted in the robot having excessively intense emotion swings. We will have to conduct further tests to evaluate the reasons behind the lack of satisfactory results in this study.

Regarding the second study (Section 5.3), after analysing the ratings measured for each dimension of the RoSAS questionnaire and the differences observed between conditions, we can conclude that there was a significant increase in how warm the participants perceived the robot to be under the *expressive* condition when compared with the *neutral* condition. While we can observe differences in the ratings for the other two dimensions, none of them were shown to be significant. The fact that there was a significant variation on the participant’s perception of the robot means that our hypothesis *H2.1* was validated. Hypothesis *H2.2* was also validated because this variation was observed in the participants’ ratings of the robot’s warmth. As expected, the participants did not find the robot to be more competent due to the modulation, which validated hypothesis *H2.4*. This happened because the items in the RoSAS questionnaires that were used to compute the *competence* dimension evaluate aspects of the interaction that are more connected with the ability of the robot to satisfactorily complete the task at hand, which is something that did not change between conditions because, in both cases, Mini was able to play the game correctly. Significant differences did appear for the competence dimension when considering only the answers given by participants that expressed a mid-high or high interest in owning a robot, which could indicate that people with an interest in knowing more about robots are more sensitive to the variations in the robot’s expressiveness. Further tests would be necessary to understand the possible reasons behind this, in order to find a way to improve how the robot is perceived also by people less interested in robotics. The results did not validate hypothesis *H2.3* because there was no variation in the ratings of *discomfort* between conditions. We considered several explanations, from the possibility that other factors besides the robot’s expressiveness play a role in how participants perceive the robot (e.g., the design of the robot’s appearance) to the design of the modulation used under the *expressive* condition (i.e., if the modulation is too extreme, it might be perceived as unnatural). However, we would need to conduct further evaluations to test if any of these explanations are true.

There is one last conclusion that we have drawn when comparing the results obtained for both experiments. It appears that adapting the expressiveness of the robot to the context of the interaction played an important role in enhancing the perception that the user had of the robot. While in both experiments, we used robots able to merge symbolic and spontaneous communication, only the second used contextual information (the state of the game that the robot and the user were playing) to adapt these components. This resulted in significant differences being perceived between the *neutral* and *expressive conditions*. It would be interesting to evaluate in other scenarios the extent of the importance that a proper adaptation of the expressiveness to the context of the interaction has for how the robot is perceived, to ensure that this observation was not an isolated case.

### 5.5. Limitations of Our Approach

While the evaluations that we conducted showed that the proposed expressiveness architecture can be successfully integrated in a social robot, some limitations remain that require further development. First, video-based evaluations can provide less accurate evaluations than real environment evaluations. This is because real interactions allow the user to observe the system directly in a real environment, providing a more truthful representation of the target application and allowing participants to appreciate aspects that might not be perceived in videos. In addition, expressiveness generation is highly platform-specific because it depends on the available output interfaces. This can make it difficult to establish a comparison with other approaches based on objective metrics. This is the reason why we chose to conduct subjective evaluations.

The other limitations are tied to the modulation approaches that we designed. On the one hand, the parameter-based modulation is designed to be controlled by the robot’s applications, which would force the developers to manually set the values for the parameters if a modulation is desired. It would be interesting to enhance the Expression Manager with the ability to control the modulation parameters to automatically adapt the robot’s expressiveness to different factors. In addition, the profile-based modulation currently only allows for affect expression, and the integration of this strategy has been performed ad hoc for combining emotions and moods. To improve our system, we need to generalise this for other internal states. Finally, although having developers handcraft the modulation profiles makes sense for our approach due to the limited amount of affective states that we considered, this could become an issue if the number of internal states increases.

Regardless of these limitations, the results extracted from the evaluations conducted show that the proposed expressiveness architecture can be used in a real robot for real-world tasks and that the proposed modulation strategies can convey the robot’s internal state in a perceivable way and can improve the perception that users have of the robot.

## 6. Conclusions

In this paper, we presented an expressiveness architecture for social robots. In our architecture, gestures are modelled using state machine-like structures, which simplifies the design of new gestures and allows us to use graphical tools to avoid having to program the expressions by hand. The Expression Manager is the module in our software architecture that controls the execution of behaviours. This is divided into four elements: the Expression Scheduler, the Expression Executor, and the Interface Players. The Scheduler controls the temporal planning of expressions and manages the conflicts that can arise between these gestures. The Executor loads the appropriate gesture from the library and controls its execution. Finally, the Players are the modules that interact directly with the output interfaces of the robot.

To avoid some of the drawbacks of using a library of predefined gestures (i.e., the need for multiple versions of each expression if we want to convey multiple states of the robot, and the lack of variability in the robot’s interactions), we integrated in our system a series of modulation approaches to provide the system with a higher degree of flexibility. The first method allows the applications of the robot to modify the content of a gesture at runtime (e.g., the sentence uttered or the final position of a movement). The second method allows modifying the general behaviour of an expression through the modulation of two parameters: speed and amplitude. Finally, the last approach uses handcrafted modulation profiles that affect those parameters of the output interfaces that are not explicitly used by the gestures, which allows the robot to express different internal states. Besides these modulation strategies, we also integrated into our expressiveness architecture two paraphrasing strategies, and a module for automatically selecting the non-verbal expressions that should accompany the robot’s speech. The first paraphrase strategy aims at adding general variability to the robot’s speech, while the second seeks to adapt these utterances to the profile of the user interacting with the robot.

The main contributions of the work presented in this paper are as follows. First, we proposed a model to design a robot’s expressiveness that roots itself in communication theory for conveying the symbolic and spontaneous dimensions of communication. While combining both dimensions is something that has been performed by other authors, our approach decouples them. This allows developers to focus on designing the symbolic dimension (i.e., the messages that seek to achieve a communicative goal), while the system automatically controls the spontaneous dimension (although developers can also tune how this dimension is conveyed). The proposed system allows us to use multimodal communication for conveying both dimensions and is able to fuse multiple internal states (currently, it has been tested with mood and emotion fusion). The second contribution is the proposal of an expressiveness framework that relies on state machines to create robotic expressions, and that integrates different techniques for adapting the robot’s expressiveness to different factors, like the context of the interaction or the identity of the user. Finally, this work also presents a series of experiments that aim to evaluate the effect that the combination of the symbolic and spontaneous dimensions of communication have on how people perceive a social robot. The proposed approach has been integrated in a robotic platform and tested in real-world applications, such as entertainment applications or cognitive stimulation exercises and therapies, among others.

While the results obtained with the proposed approach are encouraging, there is still much work that needs to be performed. First, all the experiments presented in this work were based on participants observing a single interaction with the robot. While this gives us an idea of how a new user perceives the robot, these perceptions will evolve when engaging in long-term interactions. It would be beneficial to conduct evaluations within this time frame to measure how our system performs after the users become accustomed to interacting with Mini. Also, although our system allows for simultaneous execution of expressions, it does so by simply ensuring that the expressions that will be executed at the same time will not try to use the same interfaces (which would lead to a conflict). An improved version of this method that checks for other factors (e.g., if the communicative messages transmitted by the expressions are compatible or not) would help to enhance the robot’s expressiveness. In addition, the proposed modulation techniques for conveying the spontaneous dimension of communication could benefit from improvements, for example adding mechanisms to adapt the value of the parameters to the robot’s internal state in the parameter-based approach or adding more possible states to the profile-based strategy, on top of mood and emotions. Finally, given that this approach relies on handcrafted expressions, it would be desirable to keep simplifying the process for creating new expressions to ensure that it takes away as much work from the developers as possible, while still giving them control over how these expressions look.

## Figures and Tables

**Table 1:** Effects that the modulation parameters have on each type of interface. An increased value of the parameters means an increased value for the features shown in the table, while a decrease in speed and amplitude means a decreased value for these features.

	Speed	Amplitude
**Voice**	Prosody rate	Volume and pitch
**Joints**	Motion speed	None
**LED**	Blinking speed	Brightness
**Eyes**	Blinking speed	None

**Table 2:** Differences in the robot expressiveness for the “neutral” mood and the “sad” emotion.

		Neutral	Sad
	**Expression**	Neutral	Sad
**Eyes**	**Blink frequency**	Normal	Very low
	**Gaze direction**	Looking front	Looking down
	**Pitch**	Medium-Low	Very low
**Voice**	**Prosody rate**	Normal	Slowed down
	**Volume**	Normal	Slightly low
	**Speed**	Medium-Low	Low
**Body**	**Acceleration**	Medium-Low	Low
	**Body posture**	Head looking forward, arms to the side of the body	Head looking down, arms slightly backwards
	**Colour**	Green	Blue
**Heart**	**Intensity**	Medium	Medium-low
	**Heart rate**	Normal	Slightly slowed down

## Data Availability

The datasets generated during and/or analysed during the current study are not publicly available due to the confidentiality agreements signed with the participants in the study, where it was stated that the data would not be shared with third parties, but are available from the corresponding author upon reasonable request.
